# Efficacy and safety of immune checkpoint inhibitors in recurrent or metastatic head and neck squamous cell carcinoma: A systematic review and meta‐analysis of randomized clinical trials

**DOI:** 10.1002/cam4.6564

**Published:** 2023-10-10

**Authors:** Shoutao Dang, Shurong Zhang, Jingyang Zhao, Xinyu Li, Wei Li

**Affiliations:** ^1^ Cancer Center, Beijing Tongren Hospital Capital Medical University Beijing China

**Keywords:** chemotherapy, head and neck squamous cell carcinoma, immune checkpoint inhibitors, meta‐analysis, systematic review

## Abstract

**Background:**

Immune checkpoint inhibitors (ICIs) showed antitumor activity for recurrent or metastatic head and neck squamous cell carcinoma (R/M HNSCC). However, the results from different studies were controversial.

**Methods:**

Online databases were searched for randomized clinical trials (RCTs) evaluating ICIs for R/M HNSCC. The characteristics of the studies and the results of overall survival (OS), progression‐free survival (PFS), objective response rate (ORR), treatment‐related adverse events (TRAEs) were extracted.

**Results:**

A total of 4936 patients from eight studies were included. Anti‐PD1/PDL1 monotherapy significantly improved OS in total population (hazard ratio, HR, 0.87, 95% CI, 0.79–0.95, *p* = 0.003) and PD‐L1 high expression patients (HR, 0.71, 95% CI, 0.55–0.90, *p* = 0.006) with significant lower incidence of any grade TRAEs (odds ratio, OR, 0.16, 95% CI, 0.07–0.37, *p* < 0.00001) and Grades 3–5 TRAEs (OR, 0.18, 95% CI, 0.10–0.33, *p* < 0.0001) compared with standard of care (SOC); however, the pooled results of PFS and ORR were not significant different. PD1/PDL1 inhibitors plus CTLA4 inhibitors did not improve OS, PFS, ORR compared with SOC or ICIs monotherapy; however, the incidence of Grades 3–5 TRAEs was significant higher compared with ICIs monotherapy (OR, 1.80, 95% CI, 1.34–2.41, *p* = 0.0001).

**Conclusions:**

Anti‐PD1/PDL1 monotherapy could improve OS for R/M HNSCC with significant lower incidence of TRAEs compared with SOC. PD1/PDL1 inhibitors plus CTLA4 inhibitors showed no more benefit compared with both SOC and ICIs monotherapy, but the incidence of Grades 3–5 TRAEs was significant higher compared with ICIs monotherapy.

## 1 INTRODUCTION

Head and neck squamous cell carcinoma (HNSCC) consist of tumors arising from oral cavity, pharynx, larynx, and paranasal sinuses. There were more than 743,000 new cases of HNSCC in the world annually, and it caused about 364,000 deaths during the same time period.^1^ Recurrent or metastatic (R/M) HNSCC is one of the major causes of mortality. The prognosis of R/M HNSCC is poor, with a median survival time of less than 1 year.^2^

The initial first line systemic treatment for R/M HNSCC was cisplatin based chemotherapy (cisplatin/5‐FU).^3^ In 2008, a phase III randomized trial (EXTREME) found that the addition of cetuximab to the cisplatin/5‐FU regimen improved response rate and median survival.^4^ Then the EXTREME regimen became the preferred first line regimen for the R/M HNSCC in the following decade.

Immune checkpoint inhibitors (ICIs) have been proved to be beneficial in varieties of tumors in the last decade.^5^ For R/M HNSCC, nivolumab and pembrolizumab were firstly evaluated in the platinum‐refractory setting, and the overall survival (OS) were both prolonged.^6^, ^7^ Subsequent study evaluating ICIs in the first line setting suggested that pembrolizumab plus chemotherapy or pembrolizumab monotherapy in the PD‐L1 high expression patients also prolonged OS compared with EXTREME regimen,^8^ while another study with nivolumab plus ipilimumab had negative result.^9^ Some other ICIs such as durvalumab, tremelimumab were also evaluated.^10^, ^11^ There are still growing number of related researches, but the best strategy for ICIs in R/M HNSCC patients is still indeterminate.

We conducted a meta‐analysis of all RCT studies, and comprehensively evaluated the efficacy and safety of ICIs in the treatment of R/M HNSCC.

## 2 MATERIALS AND METHODS

### 2.1 Data sources and search strategy

Relevant studies were searched from PubMed, Embase and Cochrane library up to June 31, 2023. We used the Medical Subject Headings (MeSH) terms and their entry terms as follows: “immune checkpoint inhibitors OR ipilimumab OR nivolumab OR PD‐L1 Inhibitor OR programmed death‐ligand 1 inhibitor OR CTLA‐4 inhibitor OR cytotoxic T‐lymphocyte‐associated protein 4 inhibitor OR PD‐1 inhibitor OR programmed cell death protein 1 inhibitor OR pembrolizumab OR cemiplimab OR camrelizumab OR atezolizumab OR durvalumab OR avelumab OR tremelimumab” AND “squamous cell carcinoma of head and neck OR mouth neoplasms OR laryngeal neoplasms OR oropharyngeal neoplasms OR hypopharyngeal neoplasms OR HNSCC OR SCCHN”.

### 2.2 Inclusion and exclusion criteria

This meta‐analysis followed the PRISMA (Preferred Reporting Items for Systematic Reviews and Meta‐analyses) statement.^12^

#### 2.2.1 Inclusion criteria

The inclusion criteria were as follows: (a) study type was RCT; (b) patients with pathologically confirmed R/M squamous cell carcinoma of the oropharynx, oral cavity, hypopharynx, larynx; (c) there was at least one group that was treated with ICIs monotherapy or combination; (d) there was at least one outcome of OS, progression‐free survival (PFS), objective response rate (ORR), treatment‐related adverse events (TRAEs) was reported.

#### 2.2.2 Exclusion criteria

The exclusion criteria were as follows: (a) study type was non‐RCT; (b) patients with squamous cell carcinoma of nasal cavity, paranasal sinuses, nasopharynx, cutaneous; (c) the intervention group was treated with ICIs plus radiotherapy; (d) the study data could not be extracted directly or indirectly; (e) the study was not published in English; (f) conference abstracts, case reports, comments, reviews, animal studies, and mechanistic studies.

### 2.3 Quality assessment and data extraction

The risk of bias for each study was assessed according to the Cochrane Collaboration's tool by two investigators (Shoutao Dang and Xinyu Li). The data were extracted by the same two investigators. The following information were included: study name, first author, publication year, trial phase, study design, sample size, key inclusion criteria, interventions and control group, outcomes of OS, PFS, ORR, TRAEs. The disagreements were discussed and consensus.

### 2.4 Statistical analysis

Statistical analysis was performed by Review Manager version 5.4 and Stata 17. The generic inverse variance was selected for the PFS and OS hazard ratio (HR) data, and dichotomous was used for ORR and TRAEs data. The heterogeneity of the studies was estimated using the *I* ^2^ statistics, and random‐effect model was used for *I* ^2^ > 50%; otherwise, the fixed‐effect model was used. Statistical significance was set at *p* < 0.05.

## 3 RESULTS

### 3.1 Study and data selection

**FIGURE 1:**
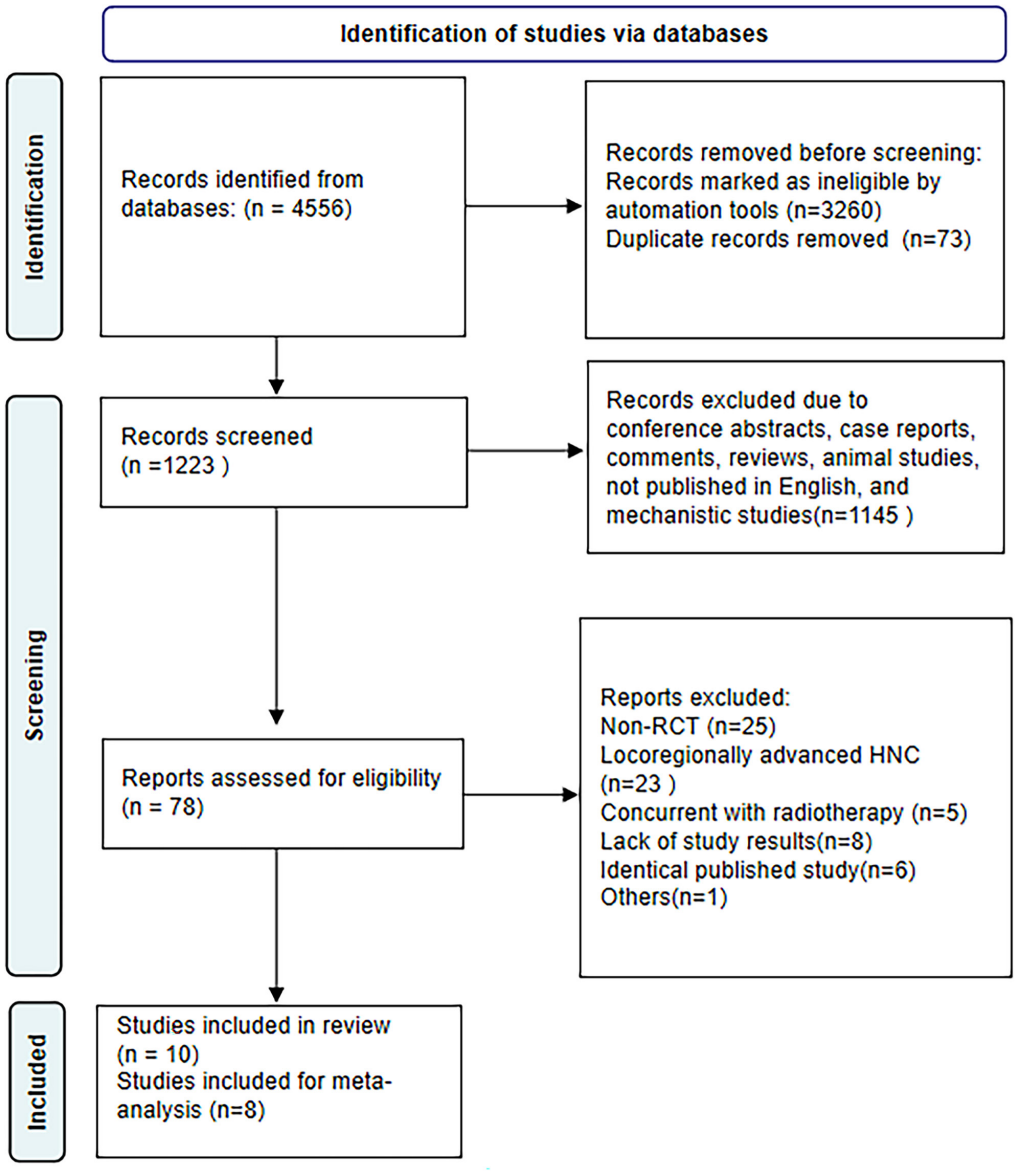
Flow diagram of the screening and selection process.

The study selection process is summarized in Figure 1. A total of 4556 studies were retrieved from the primary search strategy, of which 3260 were removed when the study type was restricted to clinical trials. And then 73 studies were excluded for duplicate records. A further 1145 studies were excluded due to conference abstracts, case reports, comments, reviews, animal studies, not published in English, and mechanistic studies. Additionally, 78 studies were assessed for eligibility, and 69 studies were excluded (non‐RCT = 25, locoregionally advanced HNC = 23, concurrent with radiotherapy = 5, lack of study results = 8, identical published study = 6, others = 1). Ten studies were included in the review. However, two studies were excluded for meta‐analysis because it could not be combined analysis with the others.^13^, ^14^ Ultimately, eight studies with 4936 patients were enrolled in this meta‐analysis.

### 3.2 Study characteristics and quality assessment

The characteristics of the included studies are shown in Table 1. Five studies^6^, ^7^, ^8^, ^10^, ^11^ compared anti‐PD1/PDL1 monotherapy with standard of care (SOC) which comprised EXTREME regimen in the first line setting and single‐agent methotrexate, docetaxel or cetuximab in the second line setting. Three studies^9^, ^10^, ^11^ compared anti‐PD1/PDL1 plus anti‐CTLA4 with SOC. Two studies compared anti‐PD1/PDL1 plus anti‐CTLA4 therapy with anti‐PD1/PDL1 monotherapy,^15^, ^16^ and the CheckMate 714 study^15^ included two different cohorts of participants as platinum‐refractory and platinum‐eligible patients. Two studies^10^, ^11^ had both monotherapy group and combination group, but these two groups were not compared directly. All the eight studies reported the incidence of TRAEs, ORR, OS, and PFS curve; however, some OS and PFS HR outcomes were not displayed. The Jayne F Tierney method was used to calculate the HR from the survival curve.^17^

**TABLE 1:** The characteristic of the eligible studies in the meta‐analysis.

Year	Study name	Study type	Key inclusion criteria	Intervention	No. of patients	outcomes
2023^11^	KESTREL	Phase 3 RCT (2:1:1)	R/M HNSCC without prior systemic therapy	Durvalumab + tremelimumab durvalumab EXTREME	823	OS, PFS, ORR, TRAEs
2023^15^	CheckMate 714	Phase 2 RCT (2:1)	Platinum refractory or platinum eligible R/M SCCHN without prior systemic therapy	Nivolumab + ipilimumab Nivolumab + placebo	425	ORR, OS, PFS, TRAEs
2022^9^	CheckMate 651	Phase 3 RCT (1:1)	Patients without prior systemic therapy for R/M SCCHN	Nivolumab + ipilimumab EXTREME	947	OS, PFS, ORR, TRAEs
2020^10^	EAGLE	Phase 3 RCT (1:1:1)	R/M HNSCC not amenable to curative therapy and platinum‐refractory	Durvalumab Durvalumab + tremelimumab SOC	736	OS, PFS, ORR, TRAEs
2019^6^	KENOTE 040	Phase 3 RCT (1:1)	R/M HNSCC and platinum refractory	Pembrolizumab SOC	495	OS, PFS, ORR, TRAEs
2019^16^	CONDOR	Phase 2 RCT (2:1:1)	PD‐L1–low/negative and platinum refractory in the R/M setting	Durvalumab + tremelimumab Durvalumab Tremelimumab	267	ORR, OS, PFS, TRAEs
2019^8^	KEYNOTE 048	Phase 3 RCT (1:1:1)	untreated locally incurable recurrent or metastatic HNSCC	Pembrolizumab Pembrolizumab + chemotherapy EXTREME	882	OS, PFS, ORR, TRAEs
2016^7^	CheckMate 141	Phase 3 RCT (2:1)	R/M SCCHN and platinum refractory	Nivolumab SOC	361	OS, PFS, ORR, TRAEs

All the eight studies were of high quality and mostly at low risk of bias, while six studies had unclear risk of allocation concealment (Figure S1).

### 3.3 Meta‐analysis of OS

**FIGURE 2:**
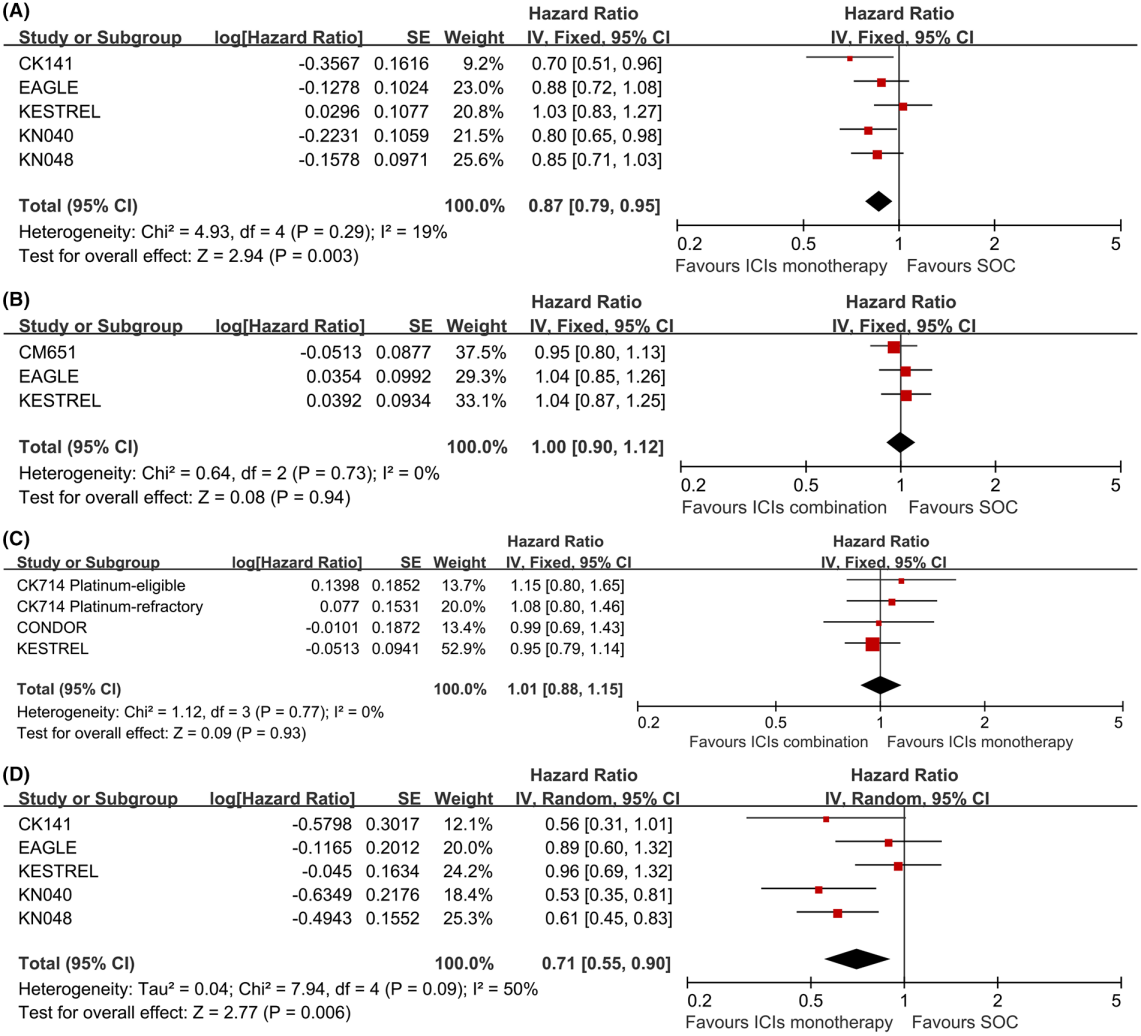
Forest plots of OS for total population in ICIs monotherapy VS SOC (A), ICIs combination VS SOC (B), ICIs combination VS ICIs monotherapy (C); for PD‐L1 high expression patients treat with ICIs monotherapy (D).

PD1/PDL1 inhibitors alone could reduce the risk of death compared with SOC (HR, 0.87, 95% CI, 0.79–0.95, *p* = 0.003, Figure 2A) in total population, while combination regimen did not reduce the risk of death compared with SOC (HR, 1.00, 95% CI, 0.90–1.12, *p* = 0.94, Figure 2B) or ICIs monotherapy (HR, 1.01, 95% CI, 0.88–1.15, *p* = 0.93, Figure 2C). We also performed exploratory analysis of OS for the PD‐L1 high expression patients. However, due to the different definitions of high expression in each study, we selected the highest PD‐L1 expression population (PD‐L1 expression level ≥ 10% in CheckMate 141, tumor proportion score ≥ 50% in KENOTE 040, combined positive score ≥ 20 in KENOTE 048, tumor cell PD‐L1 expression ≥50% or immune cells PD‐L1 expression ≥25% in KESTREL, tumor cell PD‐L1 expression ≥25% in EAGLE) for pooled analysis. ICIs alone also reduced the risk of death compared with SOC in PD‐L1 high expression patients (HR, 0.71, 95% CI, 0.55–0.90, *p* = 0.006, Figure 2D).

**FIGURE 3:**
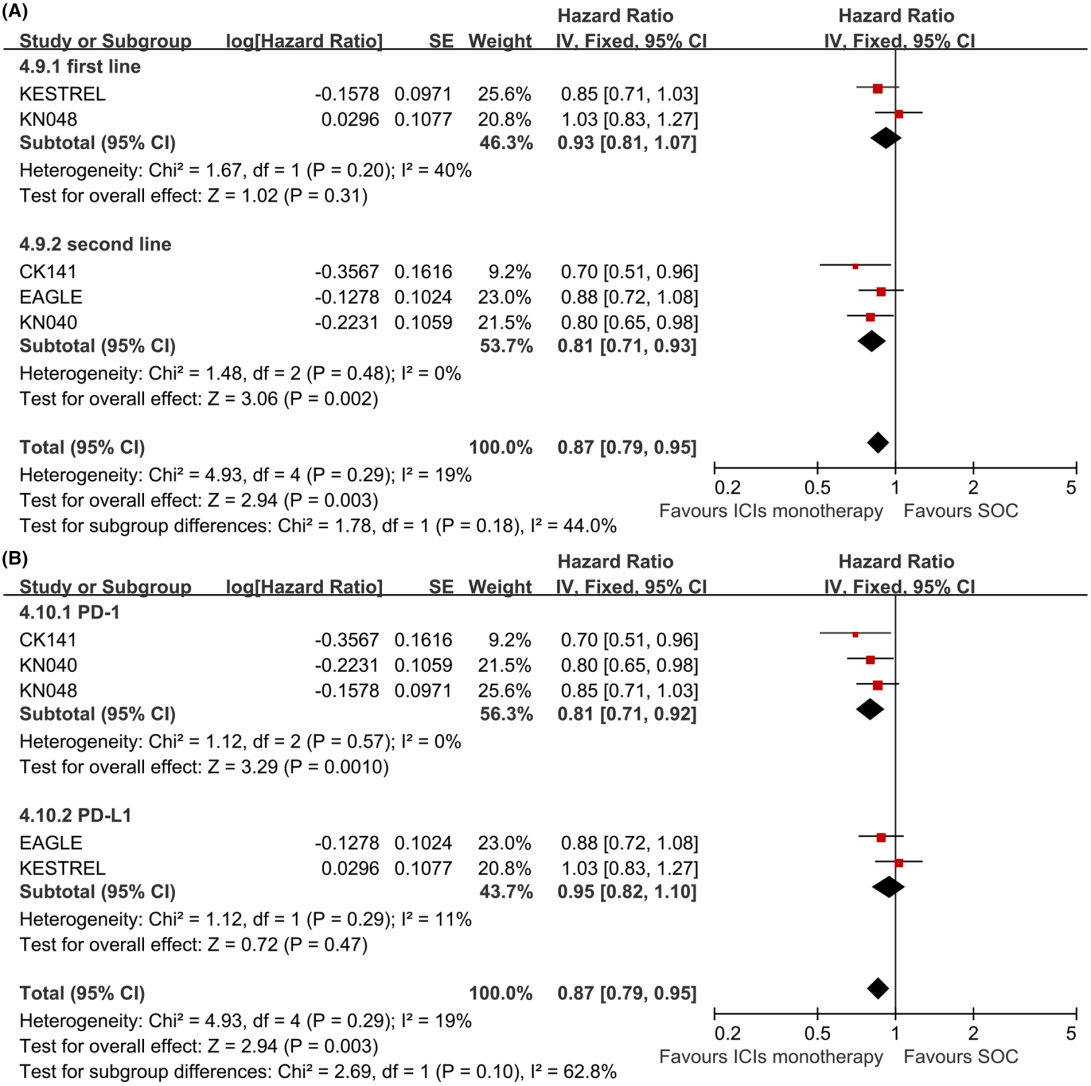
Subgroup analysis of OS for total population treated with ICIs monotherapy by therapy lines (A) and ICIs drugs used (B).

Subgroup analysis for total population treated with ICIs alone suggested that the OS was improved in the second line setting (HR, 0.81, 95% CI, 0.71–0.93, *p* = 0.002, Figure 3A) or by the anti‐PD1 drug used (HR, 0.81, 95% CI, 0.71–0.92, *p* = 0.001, Figure 3B) compared with SOC. No significant difference was found in the first line setting (HR, 0.93, 95% CI, 0.81–1.07, *p* = 0.31, Figure 3A) or for the anti‐PDL1 drug used (HR, 0.95, 95% CI, 0.82–1.10, *p* = 0.47, Figure 3B).

Subgroup analysis for PD‐L1 high expression patients treated with ICIs alone found similar results that the improvement of OS might be restricted to the second line setting (HR, 0.66, 95% CI, 0.46–0.94, *p* = 0.02, Figure S2A) or the anti‐PD1 drugs used (HR, 0.58, 95% CI, 0.46–0.73, *p* < 0.00001, Figure S2B).

### 3.4 Meta‐analysis of PFS and ORR

**FIGURE 4:**
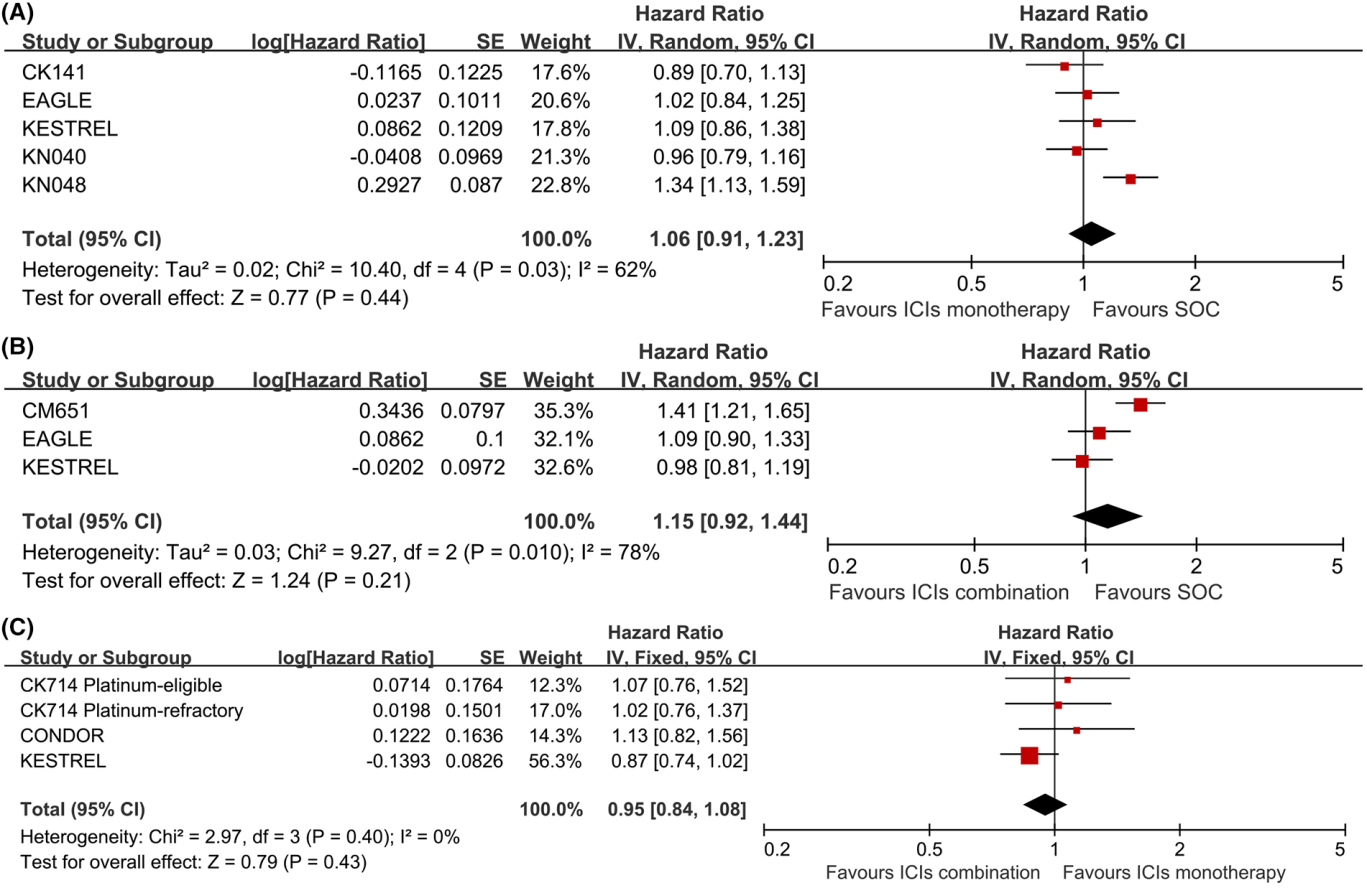
Forest plots of PFS for total population in ICIs monotherapy VS SOC (A); ICIs combination VS SOC (B) and ICIs combination VS ICIs monotherapy (C).

For total population, ICIs alone (HR, 1.06, 95% CI, 0.91–1.23, *p* = 0.44, Figure 4A) or combined with CTLA4 inhibitors (HR, 1.15, 95% CI, 0.92–1.44, *p* = 0.21, Figure 4B) did not improve the PFS compared with SOC, or for the combination therapy versus ICIs monotherapy (HR, 0.95, 95% CI, 0.84–1.08, *p* = 0.43, Figure 4C).

**FIGURE 5:**
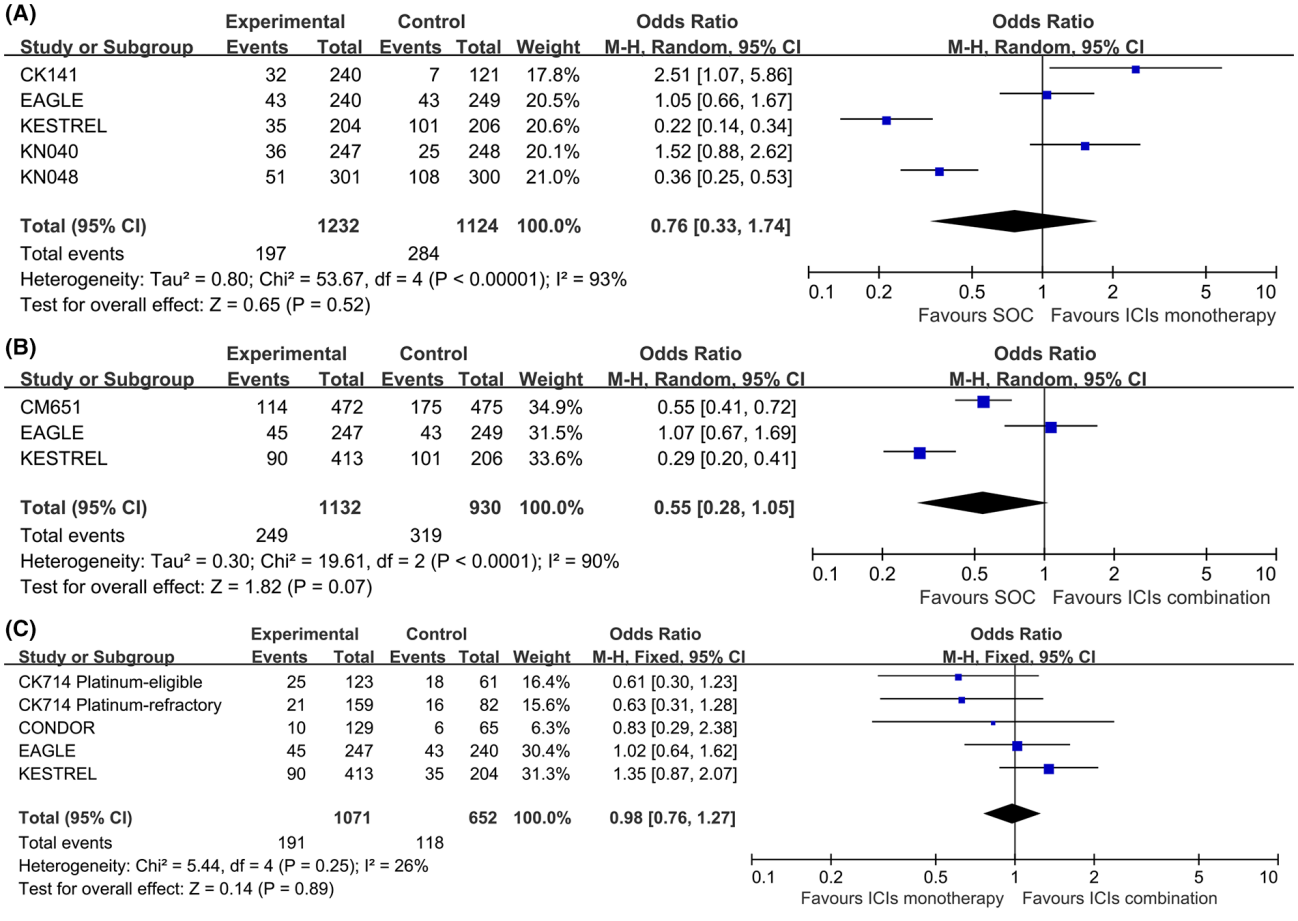
Forest plots of ORR for total population in ICIs monotherapy VS SOC (A); ICIs combination VS SOC (B) and ICIs combination VS ICIs monotherapy (C).

For the pooled ORR, no significant difference was found for ICIs alone (odds ratio, OR, 0.76, 95% CI, 0.33–1.74, *p* = 0.52, Figure 5A) or combined with CTLA4 inhibitors (OR, 0.55, 95% CI, 0.28–1.05, *p* = 0.07, Figure 5B) compared with SOC, or for the combination therapy versus ICIs monotherapy (OR, 0.98, 95% CI, 0.76–1.27, *p* = 0.89, Figure 5C). Subgroup analysis of ORR for total population by therapy lines suggested that ICIs monotherapy had significantly worse ORR versus SOC in the first line setting (OR, 0.28, 95% CI, 0.17–0.47, *p* < 0.00001, Figure S3).

### 3.5 Meta‐analysis of TRAEs

**FIGURE 6:**
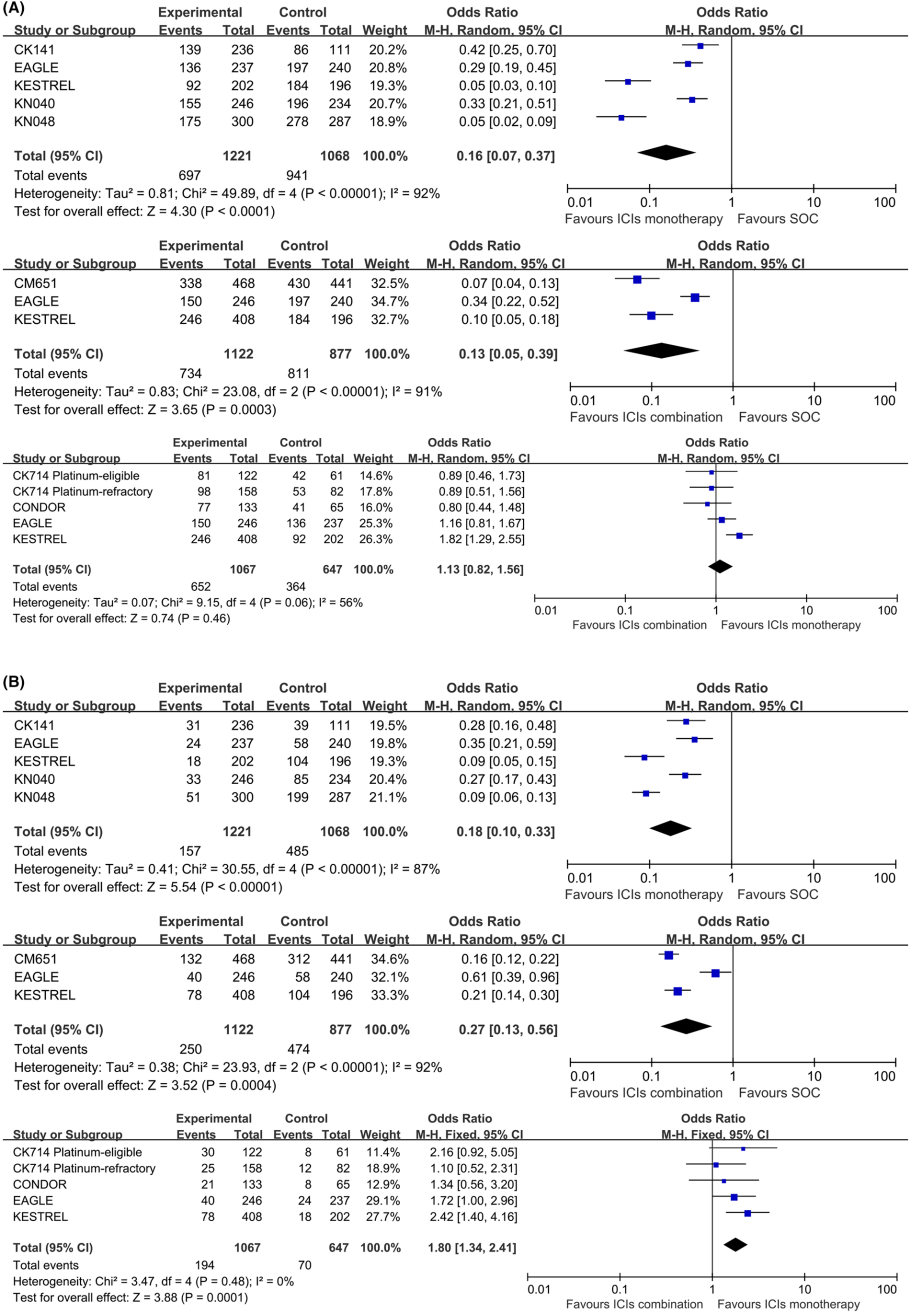
Forest plots of TRAEs for total population: any grade TRAEs (A) and Grades 3–5 TRAEs (B).

Compared with SOC, both ICIs monotherapy and ICIs combine therapy had significantly lower incidences of any grade TRAEs (OR, 0.16, 95% CI, 0.07–0.37, *p* < 0.00001; OR, 0.13, 95% CI, 0.05–0.39, *p* < 0.00001, respectively) or Grades 3–5 TRAEs (OR, 0.18, 95% CI, 0.10–0.33, *p* < 0.0001; OR, 0.27, 95% CI, 0.13–0.56, *p* < 0.00001, respectively). When compared with ICIs monotherapy, combination therapy had significantly higher incidences of Grades 3–5 TRAEs (OR, 1.80, 95% CI, 1.34–2.41, *p* = 0.0001), but not for any grade TRAEs (Figure 6).

### 3.6 Sensitivity analysis and publication bias

We conducted sensitivity analysis for the OS results in PD‐L1 high expression patients treated with PD1/PDL1 inhibitors alone. Stable result was observed by removing each trial in turn (Figure S4). Publication biases were evaluated for OS in the patients treated with ICIs monotherapy by Egger's test (total population, *p* = 0.414; PD‐L1 high expression patients, *p* = 0.587) and Begg's test (total population, *p* = 0.806; PD‐L1 high expression patients, *p* = 0.806).

## 4 DISCUSSION

R/M HNSCC has poor prognosis, and the systemic treatment options are limited. ICIs had better efficacy and safety than conventional chemotherapy or targeted therapy for R/M HNSCC; however, the appropriate strategies for individual patients are still indefinite.^18^ Our meta‐analysis indicated that ICIs monotherapy could improve the OS in total population and PD‐L1 high expression patients with significant lower incidence of TRAEs compared with SOC. To our knowledge, this is the first meta‐analysis that has pooled results of ICIs monotherapy versus EXTREME in the first line setting, anti‐PDL1 monotherapy versus SOC, PD1/PDL1 inhibitors plus CTLA4 inhibitors versus ICIs monotherapy. However, ICIs monotherapy did not significantly improve OS in the first line setting or for the anti‐PDL1 drugs compared with SOC. Furthermore, ICIs combination therapy did not improve OS, PFS, ORR compared with ICIs monotherapy, but with significant higher incidence of Grades 3–5 TRAEs.

Immune checkpoint proteins such as CTLA4 and PD1 play an important role in suppressing T‐cell activation and limiting the immune response which make tumor cells “escape” the immune surveillance.^19^, ^20^ The ICIs can “release the brake” on the immune system and promoting anti‐tumor response.^21^ This pooled analysis of five studies suggested that ICIs monotherapy could improve the OS for the total population of R/M HNSCC. This outcome consistent with a former meta‐analysis without the recent KESTREL study.^22^ We suggested that for the total population in R/M HNSCC, ICIs alone might prolong the OS compared with SOC. However, the improvement of OS might be restricted to the second line setting or the anti‐PD1 drugs used. Furthermore our meta‐analysis also found no superior and heterogeneous PFS and even worse ORR in the first line setting. Biomarkers which could predict for the ICIs were demanded to select suitable patients.

The expression of PD‐L1 was found to be associated with OS for R/M HNSCC in some studies; however, the results were still controversial.^23^ We pooled analysis for the PD‐L1 high expression patients. We found that ICIs alone also reduced the risk of death compared with SOC in PD‐L1 high expression patients. Subgroup analysis was further carried out due to the heterogeneity, and we found that the improvement of OS might also be restricted to the second line setting or the anti‐PD1 drugs used. The different results from anti‐PD1 and anti‐PDL1 drugs might be one of the reasons for the heterogeneity. And further studies are needed to estimate PD‐L1 in R/M HNSCC. Additionally, the evaluation methods for the expression of PD‐L1 and the cut‐off value varied in different studies. This might be another reason for the heterogeneity. Therefore although PD‐L1 expression is a rational biomarker for R/M HNSCC, it still has some limitations.

ICIs were considered low toxicity; however, with the increasing ICIs applied, the number of immune related adverse events (irAEs) also raised.^24^ All of the included studies reported significant lower incidence of any grade and Grades 3–5 TRAEs compared with SOC, and also for our pooled results. Nonetheless, caution should always be made because the irAEs might be delayed‐onset, permanent and even fatal.

There were also studies focused on the ICIs combine therapy. A former meta‐analysis found this combination showed no more benefit compared with SOC.^25^ Our pooled analysis found similar results that the combination regimen did not improve OS, PFS, ORR compared with SOC or ICIs monotherapy; however, the incidence of Grades 3–5 TRAEs was significantly higher in the ICIs combination group compared with ICIs monotherapy although it had better safety than SOC. Our pooled results suggested that ICIs combine therapy should be considered carefully for R/M HNSCC.

There are some limitations in this meta‐analysis. Firstly, only eight RCT studies were included, and the studies for pooled analysis in each treatment strategies were even less. Secondly, these studies involved various types of drugs and different eligible populations which might cause heterogeneity in meta‐analysis. Lastly, there was only one RCT evaluate ICIs plus chemotherapy, and none was done with ICIs plus targeted therapy although these treatment strategies were often used in the real world. High quality studies focus on this issue are urgently required.

## 5 CONCLUSIONS

Anti‐PD1/PDL1 monotherapy could improve the OS in total population and PD‐L1 high expression patients with significant lower incidence of TRAEs compared with SOC. PD1/PDL1 inhibitors plus CTLA4 inhibitors did not improve OS, PFS, ORR compared with SOC or ICIs monotherapy; however, the incidence of Grades 3–5 TRAEs was significant higher compared with ICIs monotherapy.

## AUTHOR CONTRIBUTIONS

**Shoutao Dang:** Conceptualization (equal); data curation (lead); formal analysis (lead); investigation (equal); methodology (equal); software (lead); writing – original draft (lead); writing – review and editing (supporting). **Shurong Zhang:** Conceptualization (equal); investigation (equal); methodology (equal); supervision (supporting). **Jingyang Zhao:** Conceptualization (equal); investigation (equal); methodology (equal). **Xinyu Li:** Conceptualization (equal); data curation (supporting); formal analysis (supporting); investigation (equal); methodology (equal); software (supporting). **Wei Li:** Conceptualization (equal); data curation (supporting); formal analysis (supporting); investigation (equal); methodology (equal); software (supporting); supervision (lead); writing – original draft (supporting); writing – review and editing (lead).

## FUNDING INFORMATION

No specific funding was disclosed.

## CONFLICT OF INTEREST STATEMENT

No potential conflict of interest was reported by the authors.

## Supporting information


Figure S1.

## Data Availability

The data underlying this article are available in the article and in its supplementary material.
