# Supplementary material for: Efficacy and safety of immune checkpoint inhibitors in recurrent or metastatic head and neck squamous cell carcinoma: A systematic review and meta‐analysis of randomized clinical trials
Source: Cancer Med. 2023 Oct 10;12(20):20277–86. doi: 10.1002/cam4.6564 (PMC10652313; doi:10.1002/cam4.6564)
Supplement: Supplementary file 1 — Figure S1. [file CAM4-12-20277-s001.pdf]

Efficacy and safety of immune checkpoint inhibitors in recurrent or metastatic head and neck squamous cell carcinoma: A systematic review and meta-analysis of randomized clinical trials

Shoutao Dang[1]; Shurong Zhang[1]; Jingyang Zhao[1]; Xinyu Li[1]; Wei Li[1]

Corresponding Author: Wei Li, Cancer Center, Beijing Tongren Hospital, Capital Medical University, Beijing, 100730, China. Email: weili8989@ccmu.edu.cn

[1]Cancer Center, Beijing Tongren Hospital, Capital Medical University, Beijing, 100730, China.

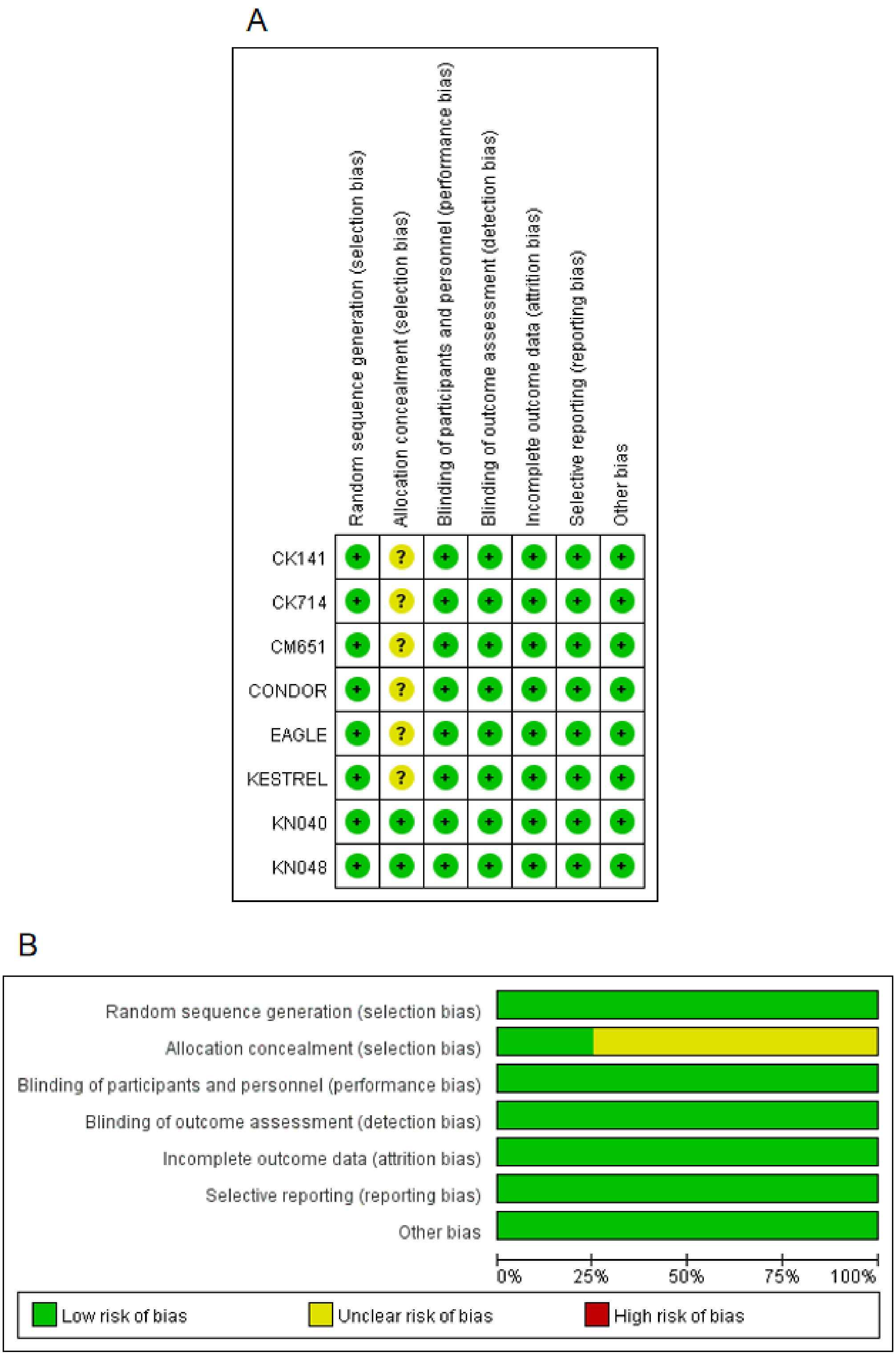

Figure S1 Risk of bias summary (A) and risk of bias graph (B)

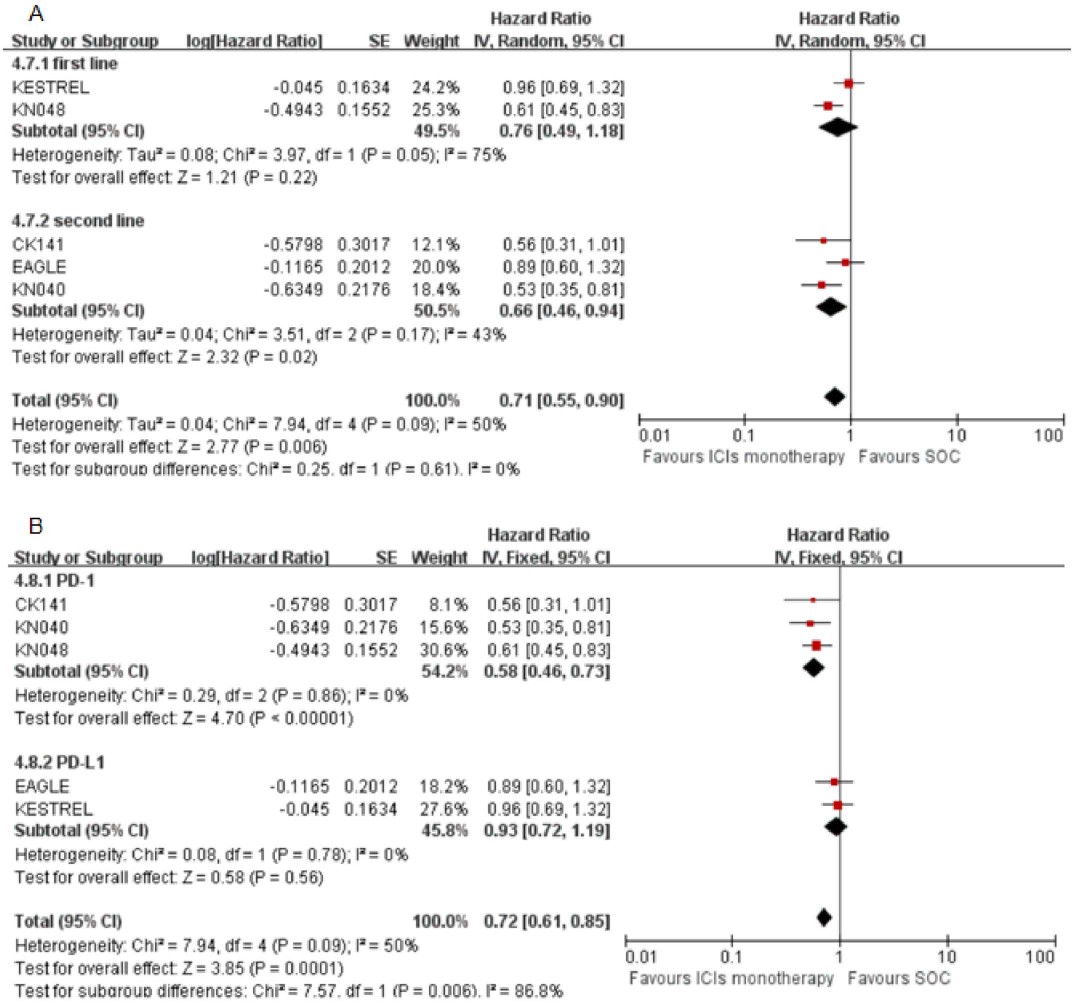

Figure S2 Subgroup analysis of OS for PD-L1 high expression patients treated with ICIs monotherapy by therapy lines(A) and ICIs drugs used (B)

Study or Subgroup	Experimental Events	Experimental Total	Control Events	Control Total	Weight	Odds Ratio M-H, Random, 95% CI
**1.32.1 first line**						
KESTREL	35	204	101	206	20.6%	0.22 [0.14, 0.34]
KN048	51	301	108	300	21.0%	0.36 [0.25, 0.53]
**Subtotal (95% CI)**		**505**		**506**	**41.6%**	**0.28 [0.17, 0.47]**
Total events	86		209			
Heterogeneity: Tau² = 0.09; Chi² = 2.96, df = 1 (P = 0.09); I² = 66%						
Test for overall effect: Z = 4.84 (P < 0.00001)						
**1.32.2 second line**						
CK141	32	240	7	121	17.8%	2.51 [1.07, 5.86]
EAGLE	43	240	43	249	20.5%	1.05 [0.66, 1.67]
KN040	36	247	25	248	20.1%	1.52 [0.88, 2.62]
**Subtotal (95% CI)**		**727**		**618**	**58.4%**	**1.43 [0.92, 2.24]**
Total events	111		75			
Heterogeneity: Tau² = 0.06; Chi² = 3.39, df = 2 (P = 0.18); I² = 41%						
Test for overall effect: Z = 1.59 (P = 0.11)						
**Total (95% CI)**		**1232**		**1124**	**100.0%**	**0.76 [0.33, 1.74]**
Total events	197		284			
Heterogeneity: Tau² = 0.80; Chi² = 53.67, df = 4 (P < 0.00001); I² = 93%						
Test for overall effect: Z = 0.65 (P = 0.52)						
Test for subgroup differences: Chi² = 22.04, df = 1 (P < 0.00001), I² = 95.5%						

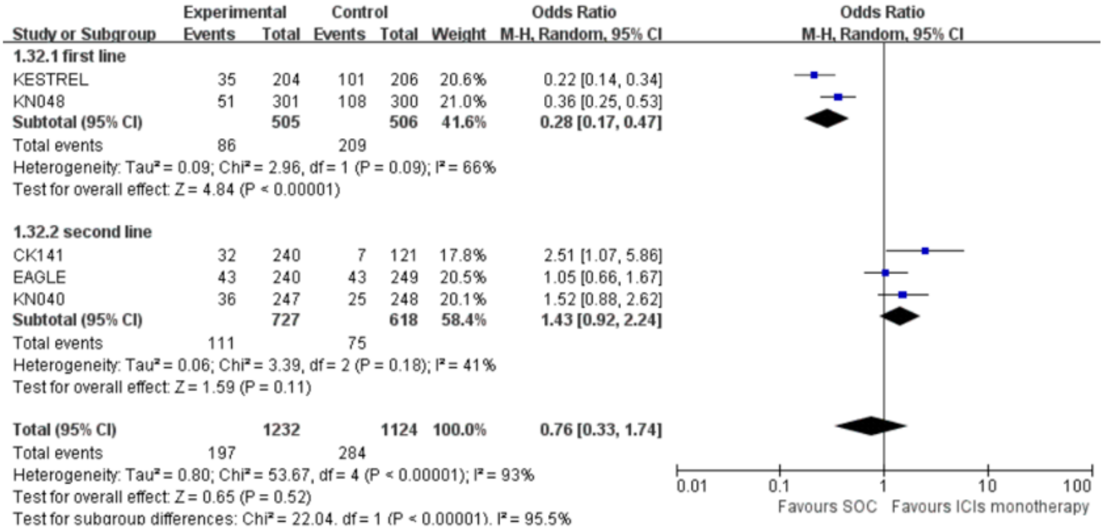

Figure S3 Subgroup analysis of ORR for total population treated with ICIs monotherapy by therapy lines

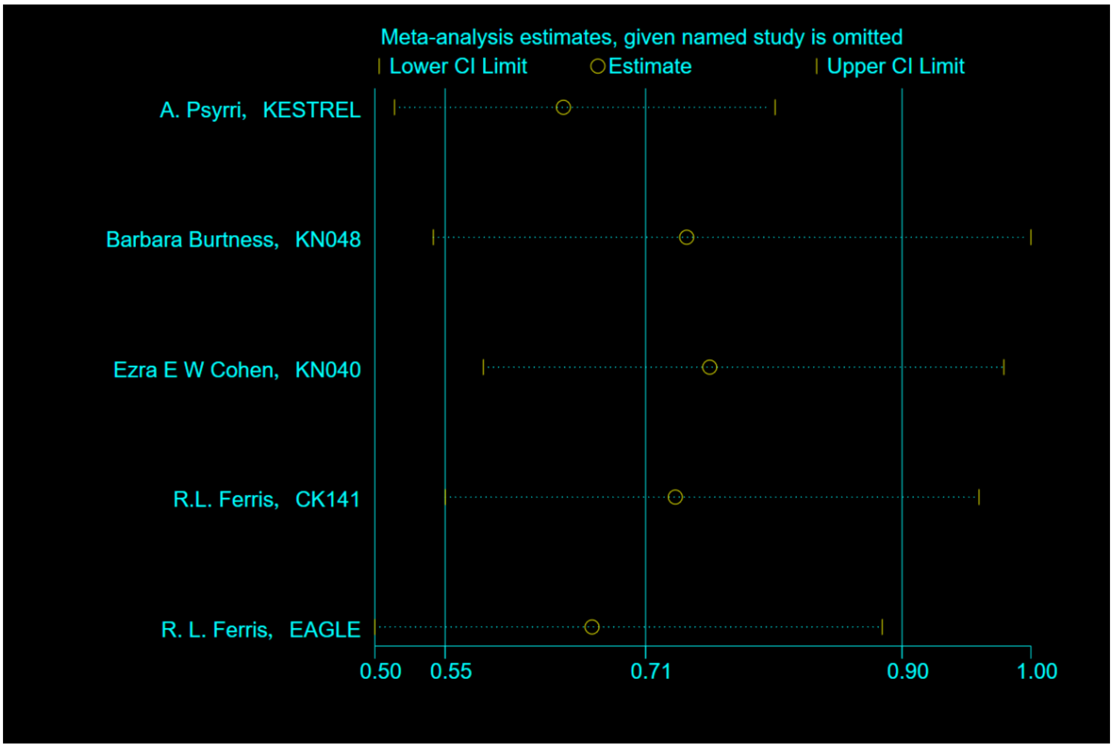

Figure S4 Sensitivity analysis of OS in PD-L1 high expression patients treated with ICIs alone.
